# Supplementary material for: Altered lipid metabolism marks glioblastoma stem and non-stem cells in separate tumor niches
Source: Acta Neuropathol Commun. 2021 May 31;9:101. doi: 10.1186/s40478-021-01205-7 (PMC8166002; doi:10.1186/s40478-021-01205-7)

Supplemental Figure Captions

Figure S1. Marker gene expression of cells in the organoid core is comparative to the cells in the corresponding pseudopalisading region of primary patient GBM. (A-D) Gene expression levels of potential regional or subtype marker genes across the TCGA, Gravendeel, Bao, and Ivy GAP datasets compared to GBM organoid regional sequencing data.

Figure S2. Oil Red O staining of GBM organoids shows lipid droplet accumulation in the organoid core region of multiple independent GBM specimens. Oil Red O staining of frozen sections of patient-derived GBM organoids at wide-field and high-power magnification (insets, representative for rim and core).

Figure S3. Oil Red O staining in primary patient tumors shows enriched lipid droplet accumulation in pseudopalisading regions of multiple independent GBM specimens. Oil Red O staining of frozen sections of patient-derived GBM organoids at wide-field and high-power magnification (insets, representative for cellular or pseudopalisading histology).

Figure S4. De novo lipid synthesis in CSCs and non-CSCs. (A-D) Incorporation of [14C]-acetate and [3H]-oleate into total phospholipids and triacylglycerol determined by liquid scintillation counting.

Figure S5. Some phospholipid classes show equal abundance in CSCs compared to non-CSCs. Quantitation of sphingomyelin (SM) lipid species from sorted CSCs and non-CSCs. No significant differences detected.

Figure S6. FADS1 and FADS2 knockdown efficiency of two independent shRNAs per gene was confirmed using qPCR. FADS1 and FADS2 gene expression levels upon shRNA knockdown compared to control shRNA.

Figure S7. Limiting dilution assays of GBM CSCs show decreased self-renewal capability upon FADS1 or FADS2 knockdown. Sphere-forming behavior of GBM CSCs upon FADS1 or FADS2 knockdown in two GBM specimens. 95% Confidence intervals are shown by dotted lines. p-values for differences in sphere forming capability were calculated pairwise by Chi-squared test.

Figure S1. Marker gene expression of cells in the organoid core is comparative to the cells in the corresponding pseudopalisading region of primary patient GBM.

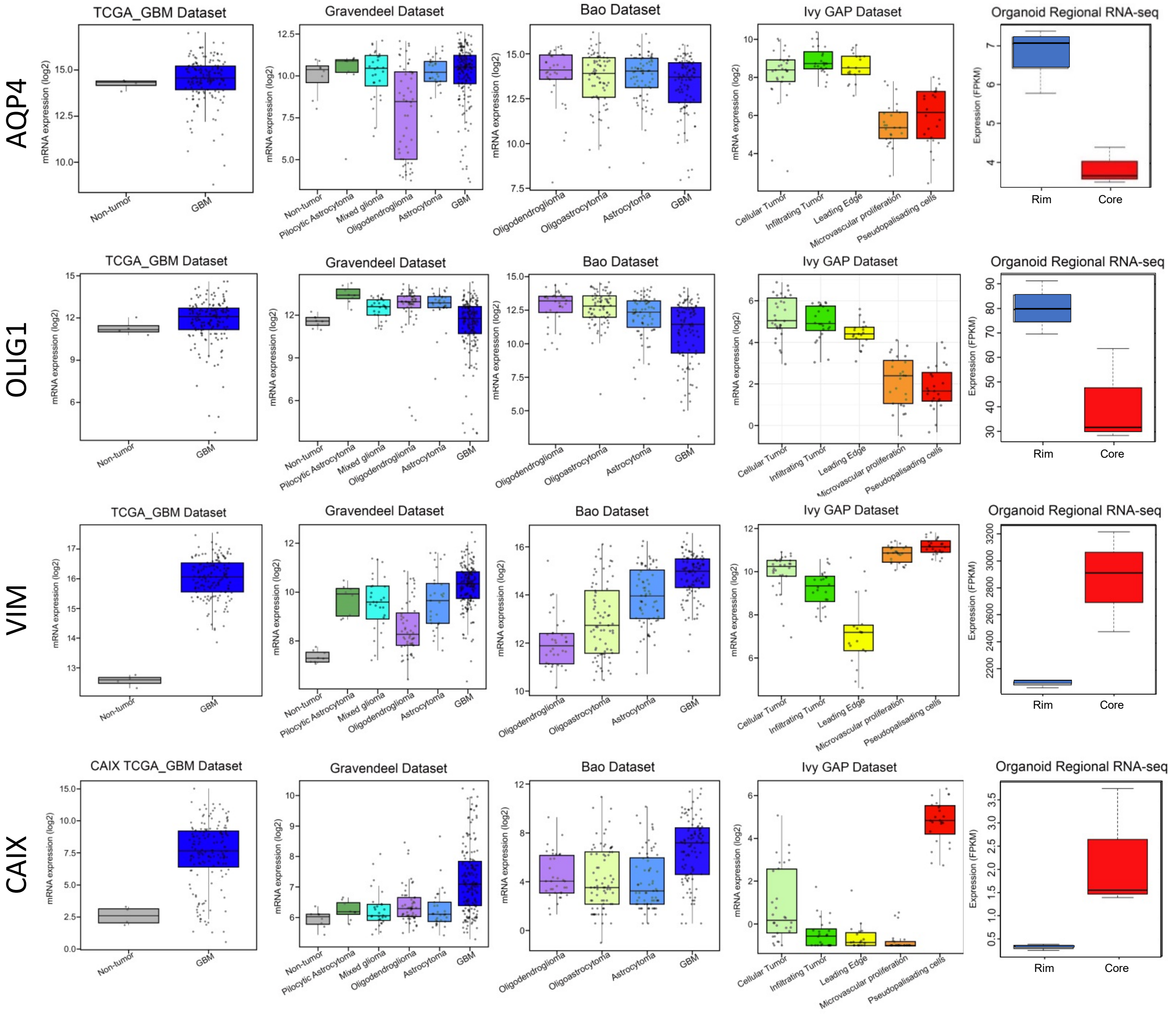

Figure S2. Oil Red O staining in 3D organoids shows enriched lipid droplet accumulation in organoid core regions.

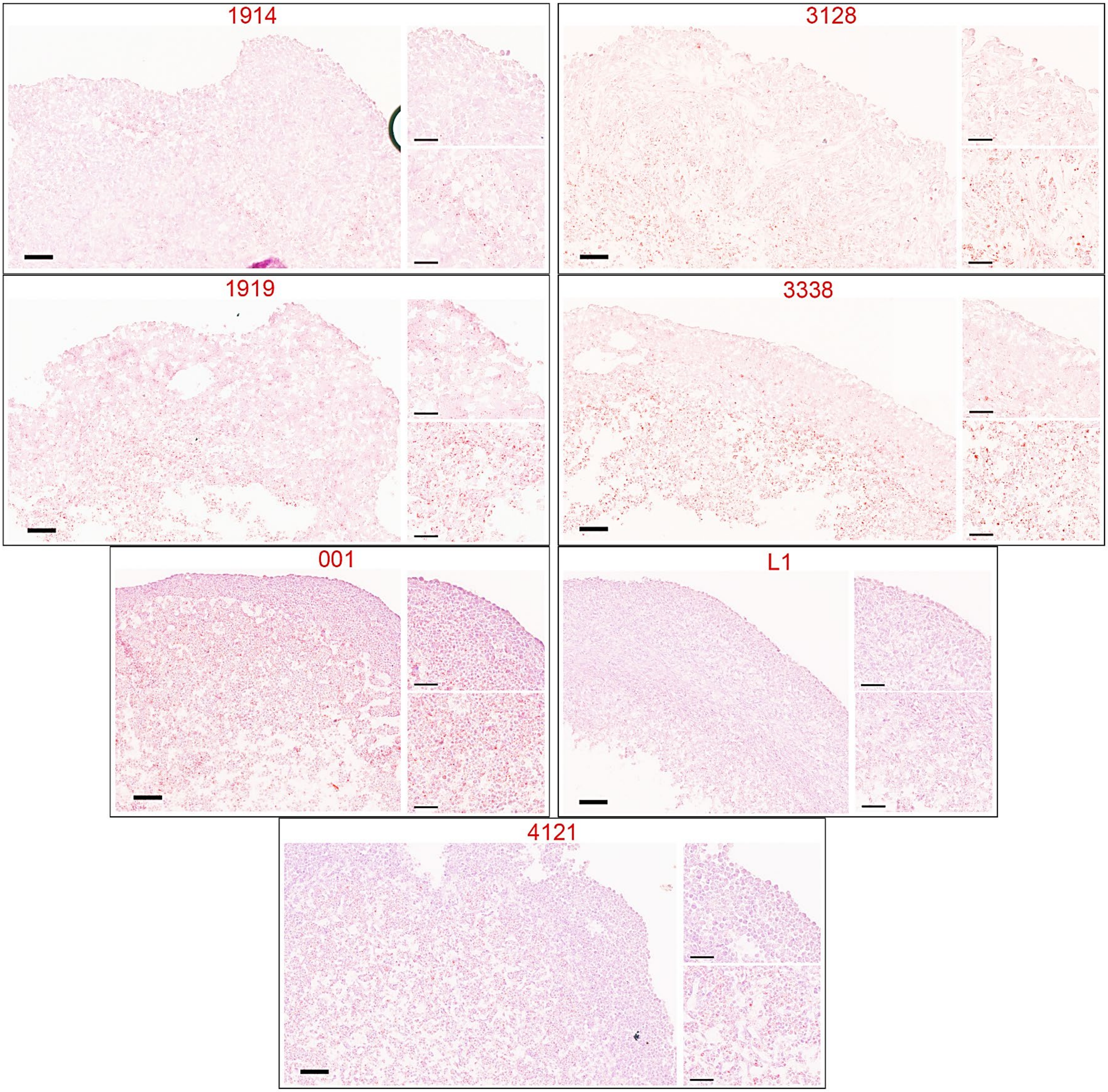

Figure S3. Oil Red O staining in primary patient tumors shows enriched lipid droplet accumulation in pseudopalisading regions.

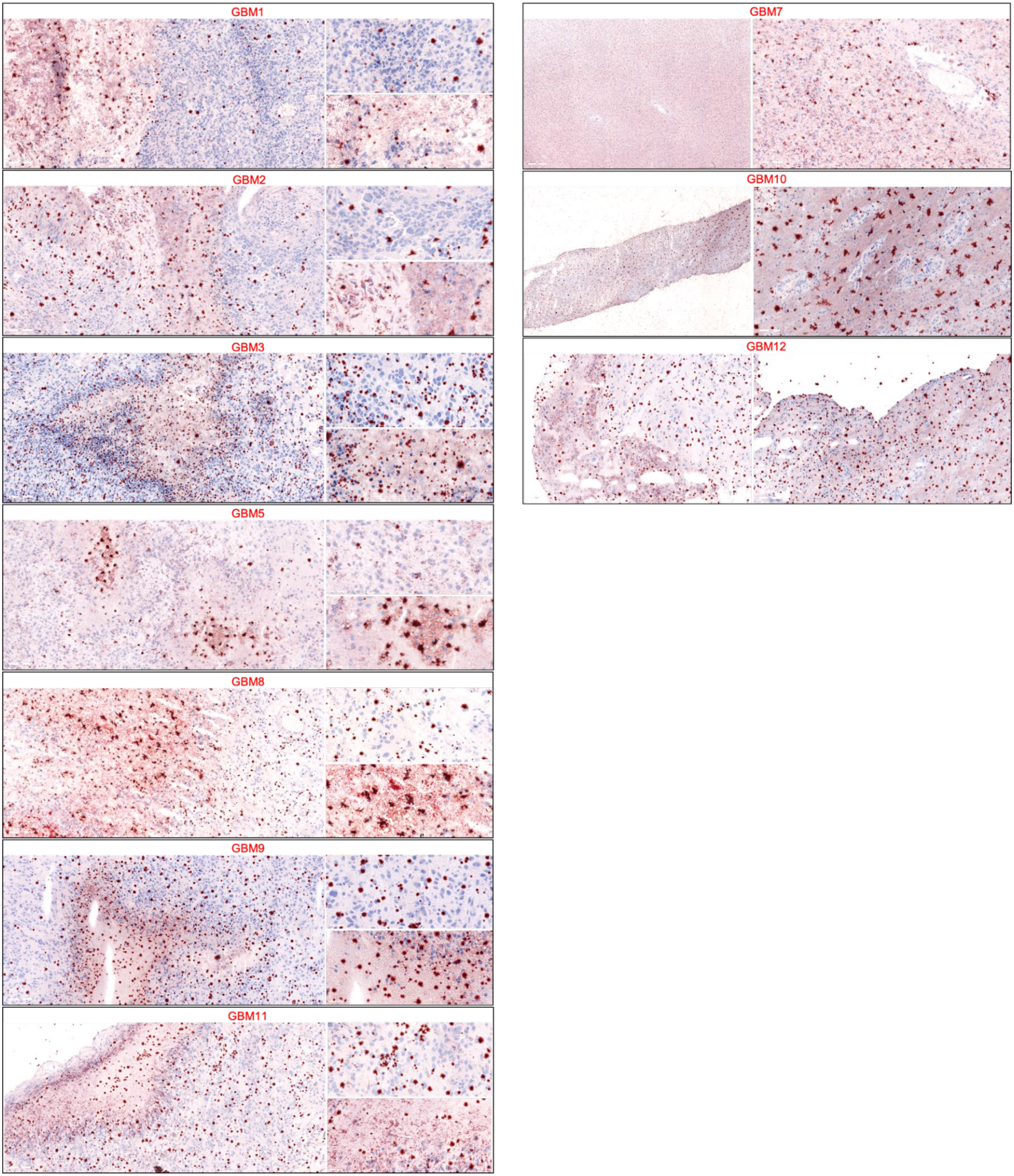

Figure S4. De novo lipid synthesis in CSCs and Non-CSCs.

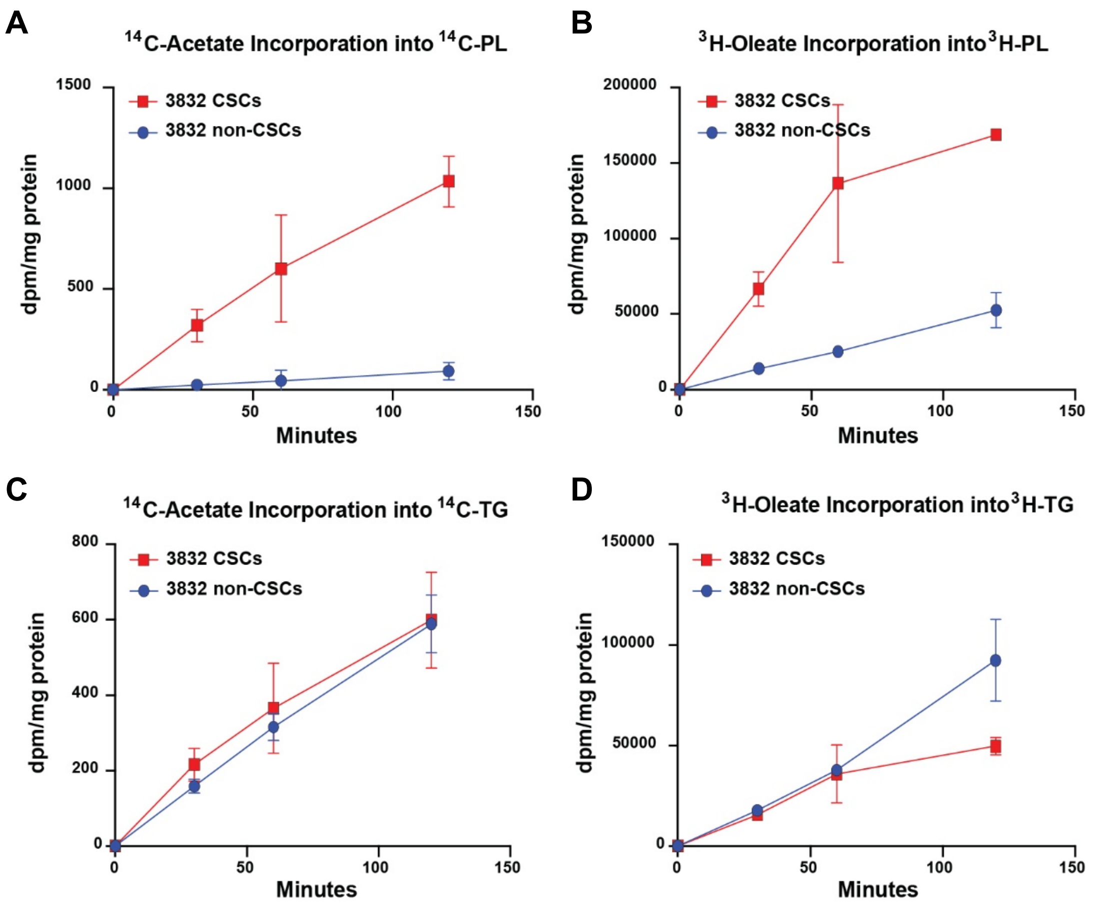

Figure S5. Some phospholipid classes show equal abundance in CSCs compared to non-CSCs.

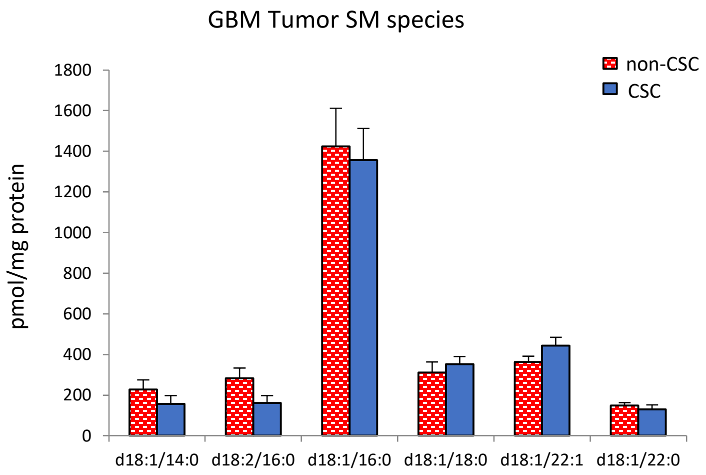

Figure S6. FADS1 and FADS2 shRNA knockdown efficiency in 3691 GBM specimen

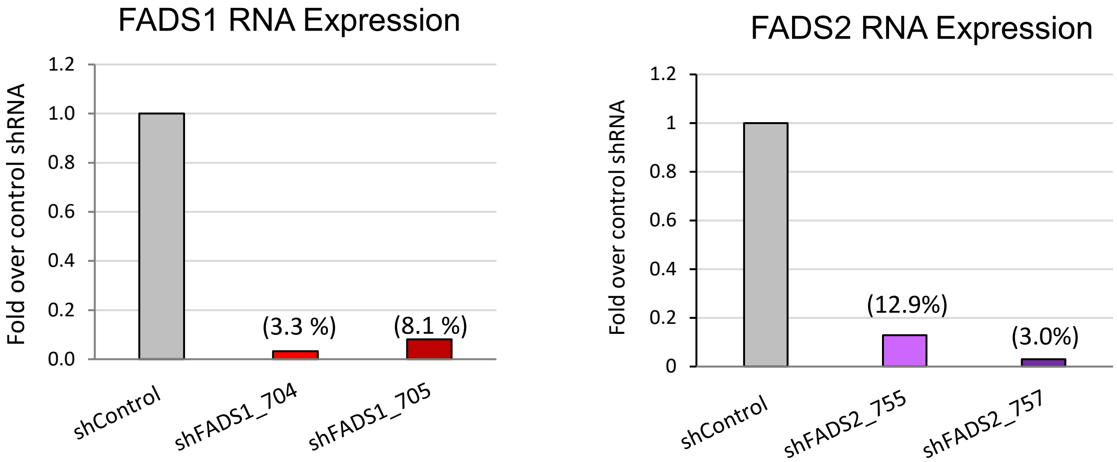

Figure S7. Limiting dilution assays to quantify self renewal in GBM CSCs with FADS1 or FADS2 knockdown.

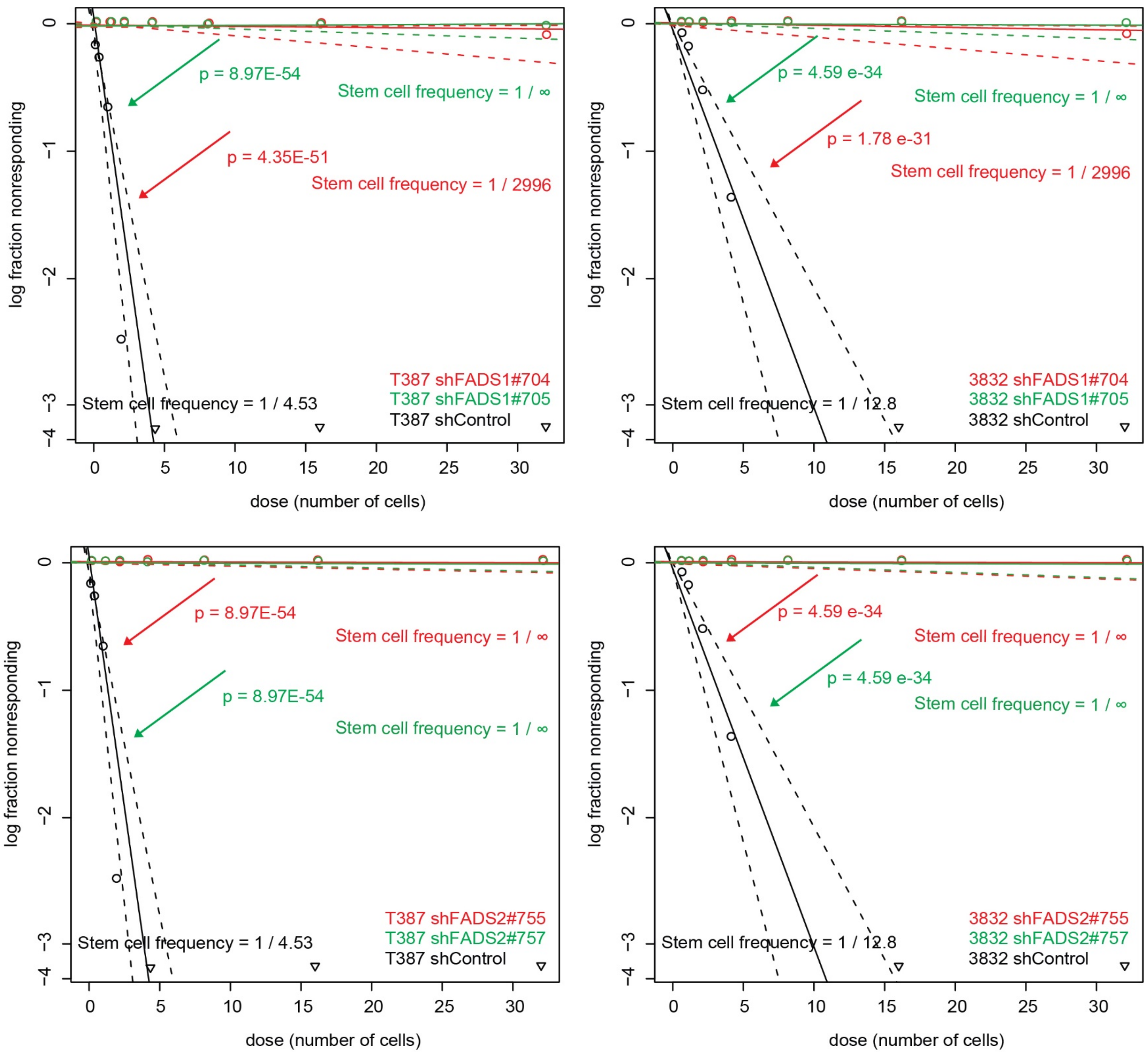

Supplement: Supplementary file 1 — Additional file 1. Supplemental Figures and Legends. [file 40478_2021_1205_MOESM1_ESM.pdf]
